# Evaluation of various cytological examinations by bronchoscopy in the diagnosis of peripheral lung cancer

**DOI:** 10.1038/sj.bjc.6601368

**Published:** 2003-11-11

**Authors:** M Kawaraya, K Gemba, H Ueoka, K Nishii, K Kiura, T Kodani, M Tabata, T Shibayama, T Kitajima, M Tanimoto

**Affiliations:** 1 Department of Respiratory Medicine, Okayama Institute of Health and Prevention, 408-1 Hirata, Okayama, Okayama 700-0952, Japan; 2 Respiratory Disease Center for Workers, Okayama Rousai Hospital, 1-10-25 Chikkoumidorimachi, Okayama, Okayama 702-8055, Japan;; 3 Second Department of Internal Medicine, Okayama University Medical School, 2-5-1 Shikata, Okayama, Okayama 700-8558, Japan

**Keywords:** rinse fluid cytology of forceps and brush, bronchoscopy, peripheral lung cancer, diagnosis, imprint cytology of biopsied sample

## Abstract

To improve the efficacy of fibreoptic bronchoscopy in the diagnosis of peripheral lung cancer, we evaluated the effectiveness of various techniques for obtaining samples for cytological examination. Between January 1984 and December 2000, flexible fibreoptic bronchoscopy under fluoroscopic guidance was performed in 1372 patients with lung cancer having no visible endoscopic findings. Histological examination of specimens obtained by forceps biopsy and cytological examinations on imprints of biopsy specimens, brushing, selective bronchial lavage, curettage, transbronchial needle aspiration, rinse fluids of the forceps, brush, curette, and aspiration needle, and all fluids aspirated during the bronchoscopic examinations were evaluated for diagnostic power. Using these techniques, the overall diagnostic rate with bronchoscopy was 93.4%. The sensitivity of the histological examination was 76.9%; additional imprint cytology increased the sensitivity to 84.8% (*P*<0.0001), while additional cytology on the rinse fluid of the forceps increased the sensitivity to 83.7% (*P*<0.0001). The addition of both imprint cytology and cytology on the rinse fluid of the forceps increased the diagnostic rate to 86.2% (*P*<0.0001). Our results indicate that cytological examinations of the imprints of biopsy samples and the rinse fluids of the forceps and the brush improve the efficacy of fibreoptic bronchoscopy in the diagnosis of peripheral lung cancer.

Fibreoptic bronchoscopy is now routinely used for obtaining specimens from which to diagnose various respiratory diseases. Bronchoscopic techniques for the diagnosis of lung cancer consist of histological examination of specimens obtained by transbronchial biopsy and cytological examination procedures such as bronchial washing, brushing, and needle aspiration. Some combinations of these techniques have been reported to increase the diagnostic sensitivity for lung cancer compared with that of transbronchial biopsy alone. Harvesting the cells shed from the tumour during bronchoscopic needle aspiration or brushing, by rinsing the needle or brush, has already been reported to be useful (Steinmann and Creul, 1978; Smith *et al*, 1980). The cytological examination of the brushing samples or bronchial washing fluid in addition to the histological examination of specimens obtained by forceps biopsy has been shown to increase sensitivity in the diagnosis of peripheral lung tumour, by an estimated 12–30% (Chaudhary *et al*, 1978; Mak *et al*, 1990; Govert *et al*, 1996). Furthermore, cytology on the rinse fluids has been previously reported to elevate the diagnostic sensitivity from 65.8 to 70.7% (Zavala, 1975; Rosell *et al*, 1998). On the basis of these results, we planned to evaluate the effects of a histological examination combined with cytological techniques such as imprints of biopsy specimens and cytology on the rinse fluids of the forceps and brush; the effects of such combinations have not been previously reported. The primary objective in this study was to evaluate the effectiveness of adding various cytological examinations to the histological examination of specimens obtained by forceps biopsy in the diagnosis of peripheral lung cancer.

## MATERIALS AND METHODS

In this study, peripheral lung cancer was defined as lung cancer that has no endoscopic findings visible by fibreoptic bronchoscopy. In our institute, every patient with peripheral lung tumour routinely underwent flexible fibreoptic bronchoscopy through the transoral route under topical 2% lidocaine anaesthesia. Transbronchial biopsy was performed under the guidance of X-ray television fluoroscopy. At least three specimens were obtained from each patient, and the imprints of biopsy specimens were prepared for Papanicolaou staining. The biopsy specimens were then fixed with 10% buffered formalin. The forceps used in the biopsy was placed in 5 ml of balanced salt solution and agitated to remove cells collected on the forceps. The bronchial brushing of the tumour was performed with a bronchial cytology brush and the material thus obtained was smeared immediately on glass slides and fixed in 95% ethanol. The brush was also placed in 5 ml of balanced salt solution and agitated.

The rinse fluids of the forceps and brush and all fluid aspirated during the fibrescopic examination were separately collected and centrifuged at 1500 r.p.m. for 2 min. The sediment was smeared on several glass slides and fixed with 95% ethanol. The cytological specimens were stained by the Papanicolaou technique. The histological specimens were stained with haematoxylin and eosin.

We performed standard histological examinations on the specimens obtained by forceps biopsy and cytological examinations on imprints of biopsy specimens, brushing specimens, the rinse fluids of the forceps and brush, and all fluid aspirated during the fibrescopic examination. When we could technically obtain neither a forceps biopsy specimen nor a brushing specimen, we tried cytological examinations on specimens obtained by curetting, transbronchial needle aspiration, or selective bronchial lavage.

McNemar's test was used to compare the sensitivities of the various bronchoscopic techniques.

## RESULTS

Between January 1984 and December 2000, 3953 patients underwent fibreoptic bronchoscopy (4089 examinations) at the Department of Respiratory Medicine, Okayama Institute of Health and Prevention. Of these patients, 1372 were ultimately given a diagnosis of peripheral lung cancer. The median age were 68 years, ranging from 29 to 90. Altogether, 962 patients were men and 410 women. The histological type of tumour was adenocarcinoma in 812 patients (59.2%), squamous cell carcinoma in 326 (23.8%), small cell carcinoma in 149 (10.9%), large cell carcinoma in 43 (3.1%), other types of carcinoma in 22 (1.6%), and unknown in 20 (1.4%).

**Figure 1:**
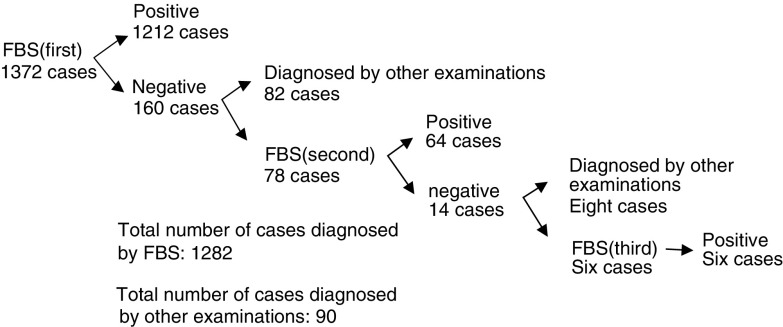
Diagnostic results of 1372 patients with peripheral lung cancer. FBS: fibreoptic bronchoscopy.

Among the 1372 patients with peripheral lung cancer, 1212 (88.3%) were given a diagnosis from the results of their first bronchoscopic examination. Furthermore, 64 patients (82.1%) of 78 undergoing the second examinations and all the six patients receiving a third examination had positive results. However, 90 patients could not receive a definite diagnosis by bronchoscopic examination. Accordingly, the overall diagnostic rate by bronchoscopy was 93.4% (Figure 1

). There were no life-threatening complications experienced. As major adverse events, we have experienced six cases of pneumothorax, and one of massive bleeding.

The diagnostic results of 1456 examinations on 1372 patients according to the bronchoscopic techniques are summarised in Table 1

**Table 1:** Diagnostic results for peripheral lung cancer by each diagnostic technique

	**No. of examinations**		
	**Evaluated**	**With positive specimen**	**Diagnostic rate (%)**
*Forceps biopsy*			
Histology	1372	1055	76.9
Imprint cytology	1368	1030	74.6
Rinse-fluid cytology of forceps	1354	919	67.9
			
*Brushing*			
Brushing cytology	1332	764	57.4
Rinse-fluid cytology of brush	1289	624	48.4
			
*Curettage*			
Curetting cytology	121	44	36.4
Rinse-fluid cytology of curette	82	21	25.6
			
*Transbronchial needle aspiration*			
Needle aspiration cytology	49	17	34.7
Rinse-fluid cytology of needle	20	7	35.0
			
All aspirated fluid cytology	1449	638	44.0
			
Selective bronchial lavage cytology	81	28	34.6

. Histological examination of specimens obtained by forceps biopsy showed the best diagnostic yield (76.9%), followed by the various cytological examinations comprising the imprints of biopsy specimens (74.6%), the rinse fluid of the forceps (67.9%), brushing specimens (57.4%), the rinse fluid of the brush (48.4%), all aspirated fluid (44.0%), transbronchial needle aspiration (34.7%), curettage (36.4%), and selective bronchial lavage (34.6%).

**Figure 2:**
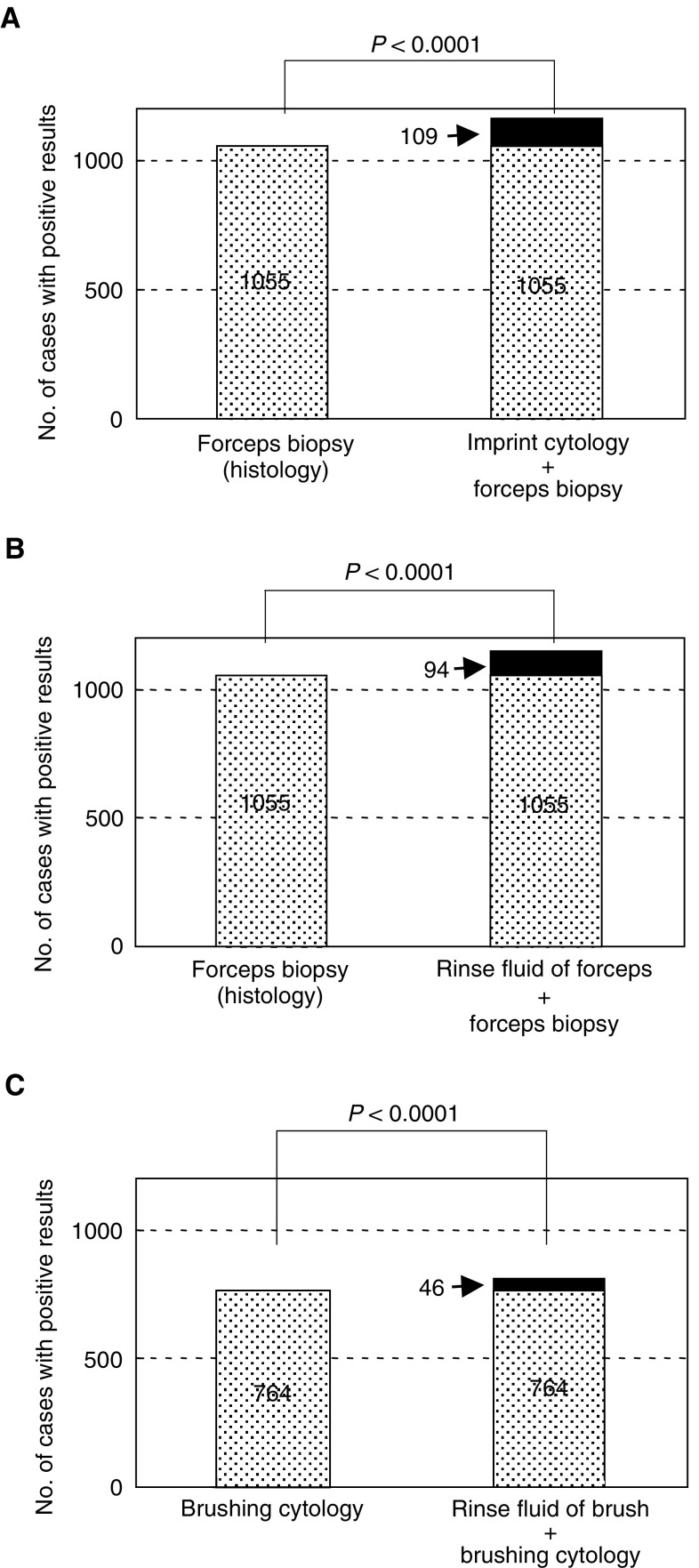
Combination effect of various diagnostic techniques. (**A**) Comparison of forceps biopsy alone with forceps biopsy plus imprint cytology. Imprint cytology of the biopsy specimens gave an additional positive result in 109 cases (7.9%). (**B**) Comparison of forceps biopsy alone with forceps biopsy plus rinse fluid cytology of forceps. The rinse fluid of the forceps gave an additional positive result in 94 cases (6.9%). (**C**) Comparison of brushing cytology alone with brushing cytology plus rinse-fluid cytology of brush. The rinse fluid of the brush gave an additional positive result in 46 cases (3.5%).

The combination effect of various bronchoscopic techniques is shown in Figure 2

. Of 1456 bronchoscopic examinations, histological specimens were obtained by forceps biopsy in 1372, and histological diagnosis was given in 1055 (76.9%). Imprint cytology of the biopsy specimens gave an additional positive result in 109 cases (7.9%). Thus, diagnostic sensitivity was significantly improved by the addition of imprint cytology (*P*<0.0001). Cytological examination of the rinse fluid of the forceps also significantly improved the diagnostic sensitivity, with additional positive results in 94 cases (6.9%) (*P*<0.0001). In 1332 bronchoscopic examinations that included brushing, cytological diagnosis was given in 764 cases (57.4%). Cytological examination of the rinse fluid of the brush gave additional positive results in 46 cases (3.5%) (*P*<0.0001).

**Figure 3:**
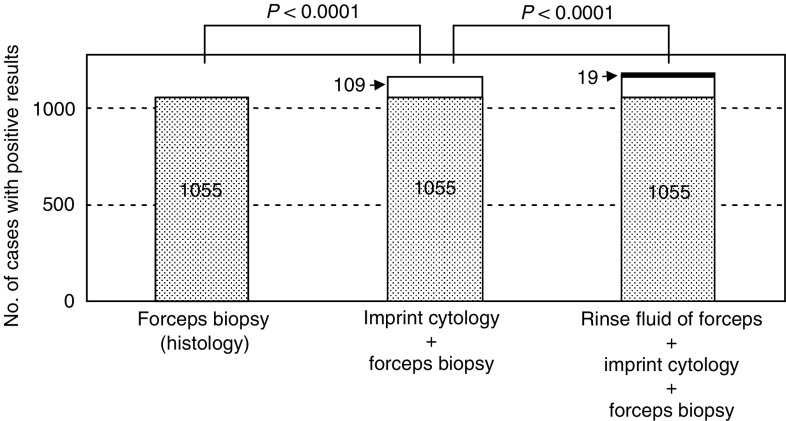
Combination effect of forceps biopsy, imprint cytology, and rinse-fluid cytology of forceps. The imprint cytology gave an additional positive result in 109 cases and the rinse fluid cytology of forceps gave further 19 cases.

The effectiveness of adding two cytological examinations, on imprints of the biopsy specimens and on the rinse fluid of the forceps, to the histological examination of specimens obtained by forceps biopsy in 1372 bronchoscopic examinations for peripheral lung cancer is shown in Figure 3

. Diagnosis was given in 76.9% of cases by biopsy alone, in 84.8% of cases by biopsy plus imprint cytology (*P*<0.0001), and in 86.2% of cases by biopsy plus imprint cytology plus rinse fluid cytology (*P*<0.0001).

Among patients who were diagnosed by bronchoscopy, 65 patients with peripheral lung cancer could not be given a diagnosis by the usual bronchoscopic techniques of forceps biopsy and brushing cytology. They were given a diagnosis by cytological examinations of one or more of the following: rinse fluid of the forceps, rinse fluid of the brush, curettage specimens, rinse fluid of the curette, needle aspiration samples, rinse fluid of the needle, all fluid aspirated during bronchoscopy, and selective bronchial lavage samples. Of these 65 patients, 27 received a diagnosis by the following single procedure alone: nine from the rinse fluid of the forceps, nine from curettage specimens, seven from needle aspiration samples, and two from the rinse fluid of the brush.

## DISCUSSION

Fibreoptic bronchoscopy has been routinely used for the diagnosis of suspected lung cancer. However, diagnostic results with bronchoscopy for peripheral lung cancer varied markedly among reports: 97.3% by Popp *et al* (1991), 74.5% by Shiner *et al* (1988), and 49.2% by Mak *et al* (1990). These differences in diagnostic results may be due to a difference of technical ability in bronchoscopic examination (Minami *et al*, 1994) or to the combination of bronchoscopic procedures for obtaining tumour specimens. In our institute, all bronchoscopic examinations were performed by three expert bronchoscopists, each with more than 15 years' experience, which may explain the high diagnostic rate in this study. Furthermore, we tried to confirm the results by cytological examinations of the obtained samples promptly at the time of bronchoscopy, and if positive results were not obtained, we repeated the examination. This may be a major reason of the good results.

We evaluated the sensitivity of transbronchial biopsy by forceps, imprint cytology of biopsy specimens, brushing cytology, cytology of the rinse fluids of the forceps and brush, and cytology of all fluid aspirated during bronchoscopic examination. In this study, we showed that the combined use of these techniques could increase the diagnostic rate to 93.4%.

Cytological examination of the rinse fluids of bronchial biopsy specimens was first mentioned by Zavala (1975). Zavala also described various diagnostic techniques performed during flexible bronchoscopy in the largest series of patients. Nevertheless, these techniques were not analysed separately, so no conclusive results could be obtained from Zavala's report. Cytological examination of the rinse fluid of biopsy specimens had not been mentioned again until 1998, when Rosell *et al* (1998) evaluated the effectiveness of this technique. They reported that this technique increased the diagnostic sensitivity of lung cancer from 65.8 to 70.7% (*P*=0.009). In the present study, we investigated the effectiveness of imprint cytology instead of the rinse fluid cytology of biopsy specimens, and found that it increased the sensitivity by 7.9 percentage points. Furthermore, we performed cytology examinations on the rinse fluids of the forceps and brush. The effects of such combinations have not been previously reported. The rinse-fluid cytology of the forceps increased the sensitivity from 76.9 to 83.8% (*P*<0.0001) and the rinse fluid cytology of the brush increased the sensitivity from 57.4 to 60.9% (*P*<0.0001). Therefore, we considered that the various cytological procedures that we used during bronchoscopy were highly useful for improving its effectiveness in the diagnosis of peripheral lung cancer.

Regarding the histological type of lung cancer, Popp *et al* (1991) reported that there was no significant difference in the diagnostic rate by histological type among brushing cytology, imprint cytology of biopsy specimens, and biopsy specimen histology. In this study, we also showed no significant difference in diagnostic rate by histological type.

Similarly, no difference in diagnostic rate by tumour size was found in this study. Kusunoki *et al* (1991) also reported that the diagnostic rate of tumours larger than 2 cm was not different from that of tumours smaller than 2 cm. Even when the target tumour is so small that it is hard to detect by chest radiographs and detectable only by chest CT scans, it is valuable to try bronchoscopic examination initially because the tumour may be visible through X-ray fluoroscopy by scanning from various directions.

The diagnostic rate of each technique was less than 80%; however, it was increased to 93.4% by combining all the techniques. Furthermore, 27 of the 1372 patients (2%) were given a diagnosis by only one technique, such as cytological examination of the rinse fluid of the forceps or brush. These results suggest that using a variety of techniques has a valuable complementary role in the diagnosis of lung cancer.

When the diagnosis for peripheral lung tumour was not obtained by the first bronchoscopic examination, the diagnostic rate was raised by repeating the examination (Figure 1). However, because bronchoscopy may be unpleasant for patients and may have a certain risk of complications, maximising the diagnostic power of bronchoscopy is important. Imprint cytology of biopsy specimens and cytological examination of the rinse fluids of the forceps and brush are safe, efficient, and inexpensive methods. By using these procedures, we will be able to avoid additional needle aspiration, CT-guided transthoracic needle aspiration, and video-assisted thoracic surgery, which are expensive and have considerable risks of complications.

In conclusion, we recommend that the inexpensive and efficient bronchoscopic techniques of imprint cytology of biopsy specimens and cytology of the rinse fluids of the forceps and brush be routinely performed in the diagnosis of peripheral lung cancer.
